# RNA interference-mediated c-MYC inhibition prevents cell growth and decreases sensitivity to radio- and chemotherapy in childhood medulloblastoma cells

**DOI:** 10.1186/1471-2407-9-10

**Published:** 2009-01-10

**Authors:** André O von Bueren, Tarek Shalaby, Christoph Oehler-Jänne, Lucia Arnold, Duncan Stearns, Charles G Eberhart, Alexandre Arcaro, Martin Pruschy, Michael A Grotzer

**Affiliations:** 1 Neuro-Oncology Program, University Children's Hospital, Zurich, Switzerland; 2 Department of Radiation Oncology, University Hospital Zurich, Zurich, Switzerland; 3 Department of Pathology, Johns Hopkins University, Baltimore, Maryland, USA; 4 Division of Clinical Chemistry and Biochemistry, University Children's Hospital, Zurich, Switzerland

## Abstract

**Background:**

With current treatment strategies, nearly half of all medulloblastoma (MB) patients die from progressive tumors. Accordingly, the identification of novel therapeutic strategies remains a major goal. Deregulation of c-MYC is evident in numerous human cancers. In MB, over-expression of c-MYC has been shown to cause anaplasia and correlate with unfavorable prognosis.

**Methods:**

To study the role of c-MYC in MB biology, we down-regulated c-MYC expression by using small interfering RNA (siRNA) and investigated changes in cellular proliferation, cell cycle analysis, apoptosis, telomere maintenance, and response to ionizing radiation (IR) and chemotherapeutics in a representative panel of human MB cell lines expressing different levels of c-MYC (DAOY wild-type, DAOY transfected with the empty vector, DAOY transfected with c-MYC, D341, and D425).

**Results:**

siRNA-mediated c-MYC down-regulation resulted in an inhibition of cellular proliferation and clonogenic growth, inhibition of G1-S phase cell cycle progression, and a decrease in human telomerase reverse transcriptase (hTERT) expression and telomerase activity. On the other hand, down-regulation of c-MYC reduced apoptosis and decreased the sensitivity of human MB cells to IR, cisplatin, and etoposide. This effect was more pronounced in DAOY cells expressing high levels of c-MYC when compared with DAOY wild-type or DAOY cells transfected with the empty vector.

**Conclusion:**

In human MB cells, in addition to its roles in growth and proliferation, c-MYC is also a potent inducer of apoptosis. Therefore, targeting c-MYC might be of therapeutic benefit when used sequentially with chemo- and radiotherapy rather than concomitantly.

## Background

Medulloblastomas (MB) are the most common malignant pediatric neoplasms of the central nervous system. MB constitute 20% of all pediatric brain tumors [1] and are characterized by their aggressive clinical behavior and a high risk of leptomeningeal dissemination. With current treatment strategies, nearly half of all patients eventually die from progressive tumors. Accordingly, the identification of novel therapeutic strategies remains a major goal.

The c-MYC proto-oncogene encodes a nuclear phosphoprotein involved in the transcription of genes central to regulating the cell cycle [2-4], proliferation [5,6], apoptosis [7-9], telomere maintenance [10,11], angiogenesis [12], and differentiation [13]. The c-MYC oncoprotein plays a pivotal role as a regulator of tumorigenesis in numerous human cancers of diverse origin [14-17]. Deregulated expression of c-MYC is often associated with aggressive, poorly differentiated tumors [4]. Deregulation of c-MYC expression is evident in MB with c-MYC gene amplification or aberrant signal transduction of wingless (WNT) signaling pathway [18]. In MB, high c-MYC mRNA expression and c-MYC gene amplification have been described as indicators of poor prognosis [19-22]. Furthermore, two studies have demonstrated that high c-MYC mRNA expression is associated with tumor anaplasia [23,24].

Developing therapeutic approaches to inhibit c-MYC would have an enormous impact on the treatment of a wide range of human cancers [25-27]. Many strategies are currently under development to target c-MYC in tumor cells, including inhibitors that block c-MYC expression, such as antisense oligonucleotides and small interfering RNA (siRNA) [28]. One aspect to be considered by evaluating the potential of c-MYC as a novel therapeutic target in MB, is its impact on cellular sensitivity to radio- and chemotherapy. In the present study, siRNA-mediated c-MYC inhibition was used to study the biological role of c-MYC in a representative panel of human MB cells expressing different levels of c-MYC.

## Methods

### Human MB cell lines

DAOY (wild-type), DAOY V11 (empty vector-transfected), and DAOY M2 (c-MYC-transfected) human MB cells have been described previously [24]. D341 and D425 human MB cells were the kind gift of Dr Henry Friedman, Duke University, Durham, NC, USA. DAOY, D341, and D425 MB cell lines are derived from three different MB obtained at craniotomy of patients aged 3–5 years [21,29,30]. All MB cells were cultured in Richter's zinc option medium (Invitrogen; Basel, Switzerland) supplemented with 10% fetal bovine serum. Non-essential amino acids were added to the medium of D341 and D425 cells to a final concentration of 1%, and G418 was added to the medium of DAOY V11 and DAOY M2 to a final concentration of 500 μg/ml. All cell cultures were maintained at 37°C in a humidified atmosphere with 5% CO_2_.

### Down-regulation of c-MYC

The Silencer c-MYC validated siRNA targeting the 3'-untranslated region of the human c-MYC mRNA sequence [31,32], referred to here as c-MYC siRNA, and Silencer scrambled c-MYC negative control siRNA, referred to here as control siRNA, having no significant homology to any known gene sequences from mouse, rat, or human, were purchased from Ambion, and used according to the manufacturer's instructions [33]. In brief, small interfering RNAs (siRNAs) for c-MYC and scrambled control siRNA were transfected at a final concentration of 30–60 nM with siPORT amine (Ambion) in the case of DAOY MB cells and with siPORT NeoFX (Ambion) in the case of D341 and D425 MB cells as described before [34,35]. Comparable with other reports we found reproducible inhibition of RNA and protein expression at 48–72 h post transfection [34,35].

### Real-time quantitative polymerase chain reaction

Total RNA isolation, reverse transcription reactions, and RT-PCR were performed as described previously [36]. Kinetic real-time PCR quantification of target genes was performed using the ABI Prism 7700 Sequence Detection System (Applied Biosystems; Rotkreuz, Switzerland), as described previously [36]. Primers and probes for c-MYC (assay ID: Hs00153408_m1) and the endogenous control 18S rRNA (assay ID: Hs99999901_s1) were purchased from Applied Biosystems (Rotkreuz, Switzerland). Primers and probes for hTERT were purchased from Microsynth (Balgach, Switzerland): forward primer, 5'-TGACACCTCACCTCACCCAC-3', reverse primer, 5'-CACTGTCTTCCGCAAGTTCAC-3', probe, 5'-ACCCTGGTCCGAGGTGTGTCCCTGAG-3'. Experiments were performed in triplicate for each data point. Relative mRNA expression of target genes was calculated by using the comparative C_T _method [37].

### Western blot analysis

The expression of c-MYC, cyclin-dependent kinase (CDK) inhibitor p21 ^(waf1/Cip1)^, caspase-9 and β-actin protein was assessed by Western blot analysis. In brief, human MB cells transfected with siRNAs for 72 h or treated with chemotherapy for 72 h after 48 h of transfection with siRNAs, were lysed with lysis buffer (1 ml/10^7 ^cells, 50 mM Tris-HCl buffer [pH 8.0], 150 mM NaCl, 1% Nonidet P40, 0.1% sodium deoxycholate, 0.1% sodium dodecylsulfate, 1 mM EDTA, and 1 mM EGTA) containing protease inhibitors (Complete, Roche; Basel, Switzerland) and incubated on ice for 30 min. After measuring the protein concentration by the BCA method (Pierce; Rockford, USA), 12 μg total protein lysates were separated by 10% SDS-polyacrylamide gels and the gels were subjected to immunoblotting. Nonspecific binding sites were blocked by 3 h incubation in TBST (10 mM Tris [pH 8.0], 150 mM NaCl, 0.05% Tween-20) supplemented with 5% non-fat dry milk. Membranes were incubated overnight at 4°C with a 1:2000 dilution of rabbit polyclonal anti-c-MYC antibody (Santa Cruz Biotechnology; Heidelberg, Germany), with a 1:1000 dilution of mouse monoclonal anti-p21 ^(waf1/Cip1) ^antibody (Upstate), and with a 1:1000 dilution of rabbit polyclonal antibody specific for cleaved caspase-9 (Cell signaling).

Membranes were then washed three times at room temperature in TBST for 30 min each time, and bound Ig was detected using anti-isotype monoclonal secondary antibody coupled to horseradish peroxidase (Santa Cruz Biotechnology; Heidelberg, Germany). The signal was visualized by enhanced chemiluminescence ECL (Amersham Biosciences; Dübendorf, Switzerland) and autoradiography. Next, immunoblotting with a 1:5000 dilution of a mouse monoclonal primary β-actin antibody (Sigma; Basel, Switzerland) was performed to verify the protein loading. For densitometry, the zymographic profiles of the gels were scanned. Relative band intensities were determined using Quantity One analysis software (Bio-Rad).

### c-MYC transcription factor binding activation assay

The binding activation of c-MYC was measured using the Mercury TransFactor assay (BD Clontech, Basel, Switzerland), an enzyme-linked immunosorbent assay (ELISA)-based assay, according to the manufacturer's instructions, as previously described [36].

### Cell proliferation

Following transfection with siRNAs, a colorimetric 3-(4,5-dimethylthiazol-2-yl)-5-(3-carboxymethoxyphenyl)-2-(4-sulfophenyl)-2H-tetrazolium inner salt (MTS) assay (Promega; Wallisellen, Switzerland) was used to quantitate cell viability of human MB cells, as previously described [36]. Each experiment was performed in triplicate. The absorbance values of each well were measured with a microplate spectrophotometer (Molecular Devices; Sunnyvale, CA) at 490 nm. All proliferation assays were repeated as independent experiments at least twice.

### Clonogenic survival assay

A clonogenic survival assay was performed using cells transfected with siRNAs for 48 h, as described previously [38]. The number of single cells seeded was adjusted to obtain around 100 colonies per cell culture dish [39]. Cells were maintained at 37°C in a humidified atmosphere containing 5% CO_2 _and allowed to grow for 9 (DAOY wt, V11, M2), 14 (D341), or 12 (D425) days respectively, before fixation in methanol/acetic acid (75%:25%) and staining with Giemsa. Only colonies with more than 50 cells were counted. All clonogenic assays were repeated as independent experiments at least twice.

### Cell cycle analysis

Cells transfected with siRNAs were harvested after 48 h by trypsinization, washed with PBS 1×, and fixed with 1 ml of 70% ethanol [38]. After washing twice in PBS, the cells were stained with a solution containing 50 μg/ml propidium iodide (Becton-Dickinson; Allschwil, Switzerland) and 100 U/ml RNase A (Qiagen; Hombrechtikon, Switzerland) in PBS for 30 min at room temperature. The percentage of cells in the different phases of the cell cycle was determined by evaluating DNA content according to methods previously described [36]. A total of 30'000 events per sample were acquired. Flow cytometric analysis was performed on a FACSCalibur flow cytometer (BD Biosciences; Allschwil, Switzerland) with CELLQuest software (BD Biosciences). The percentages of cell cycle distribution were calculated on linear PI histograms using the mathematical software ModFit LT 2.0 (Verity Software House; Topsham, ME)

### Telomerase activity

Telomerase activity in MB cell lines was measured using the Telomerase PCR ELISA kit (Roche Diagnostics; Rotkreuz, Switzerland) as previously described [40]. For this procedure, 10^6 ^cells were lysed and homogenized in 200 μl CHAPS buffer. After 30 min incubation on ice, the lysates were centrifuged at 16,000 g for 30 min at 4°C. Protein concentration was measured using the BCA method (Pierce; Rockford, USA). For the elongation step, 2 μl of cell extract (2 μg protein/μl) were incubated in 50 μl reaction mixture at 25°C for 30 min to allow the telomerase to add telomeric repeats (TTAGGG) to the end of the biotin-labeled synthetic P1-TS primer. The products extended by telomerase were then amplified by PCR in the presence of the biotin-labeled P1-TS primer and another primer, P2. Amplification products from the PCR were next immobilized in the well of a StreptAvidin-coated microplate and hybridized to a digoxigenin (DIG)-labeled probe specific for the telomeric repeat sequence. Finally, the DIG-labeled hybrids were visualized with a peroxidase-conjugated anti-DIG antibody and a colorimetric peroxidase substrate and quantified photometrically. In order to visualize the typical 6-nucleotide-ladder resulting from the TRAP assay, the amplification products from the PCR were separated by 12.5% polyacrylamide gel electrophoresis (PAGE), blotted onto a positively charged membrane, and incubated with a StreptAvidin alkaline phosphatase conjugate. The blotted products were visualized by enhanced chemiluminescence (Biotin Luminescence Detection Kit Roche Applied Science; Rotkreuz, Switzerland) and autoradiography.

### Apoptosis assay

Cell lysates were obtained from exponentially growing MB cells transfected with siRNAs for 72 h and from MB cells treated with etoposide and cisplatin for 72 h after 48 h of transfection with siRNAs. A photometric enzyme immunoassay (Cell Death Detection ELISA; Roche Diagnostics, Basel, Switzerland) was used for the quantitative determination of cytoplasmic histone-associated DNA fragments, as described previously [36]. In brief, after the different treatment schedules, cells were counted, and cell lysates of equal number of cells (5 × 10^4 ^cells/ml) were placed in a StreptAvidin-coated microtiter plate. A mixture of biotin-labeled monoclonal histone antibody and peroxidase-conjugated monoclonal DNA antibody was then added, followed by incubation for 2 h. After washing to remove unbound antibodies, the amount of mono- and oligonucleosomes was measured at 405 nm (reference wavelength 490 nm).

### Chemo- and radio-sensitivity assays

After 48 h of transfection with c-MYC siRNA or control siRNA, appropriate numbers of exponentially growing MB cells were seeded in 96-well plates. Cells were treated for 72 h with various concentrations of etoposide and cisplatin or were irradiated with 4 Gy and 10 Gy, using a Pantak Therapax 300 kV X-ray unit at 0.7 Gy/min. Dosimetry was controlled with a Vigilant dosimeter. Cell viability of human MB cells was quantified using the MTS assay 72 h after adding the chemotherapeutic substances and irradiation, respectively.

### Statistical analysis

All data are expressed as mean ± SD. Student's t-test was used to test statistical significance. *P *< 0.05 was considered to be significant. GraphPad Prism 4 (GraphPad Software, San Diego, California) software was used to calculate IC_50 _values and their 95% confidence intervals and to statistically compare the fitted midpoints (log IC_50_) of the two curves.

## Results

### Targeting c-MYC by siRNA in MB cells

**Figure 1:**
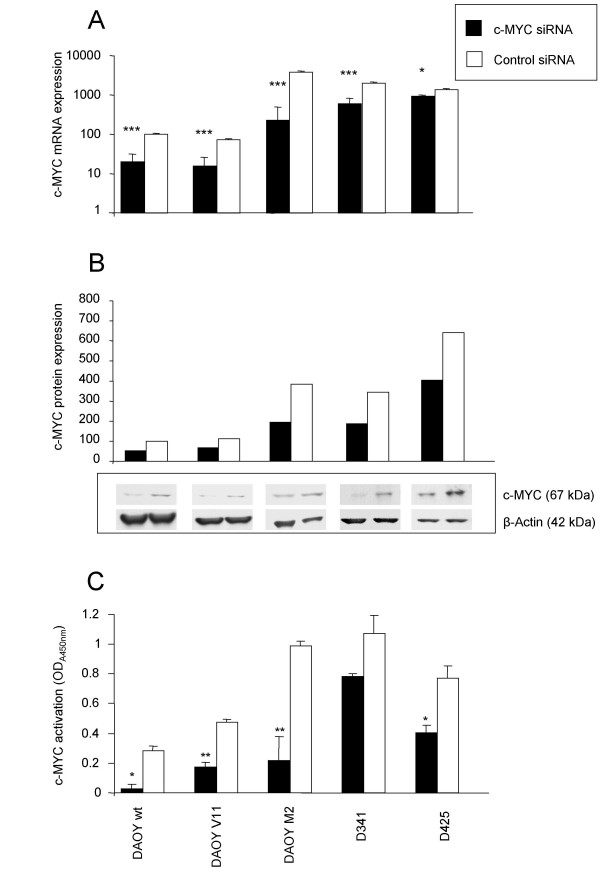
**Knockdown of c-MYC**. c-MYC siRNA reduces c-MYC mRNA **(A)**, protein **(B) **expression, and binding activity **(C) **as determined by quantitative RT-PCR, Western blot analysis, and by the ELISA-based TransAM-c-MYC binding activity assay (representative of two to three independent experiments). Values represent levels of c-MYC mRNA (n = 3; ± SD) **(A) **or protein **(B) **of control and c-MYC siRNA-transfected cells relative to control siRNA-transfected DAOY wt cells, which was set at 100 **(A, B)**. Data show the mean absorbance (n = 3; ± SD) **(C)**. * Significantly different from control values, as determined by the Student's t-test (***: P < 0.001, **: P < 0.01,*: P < 0.05).

To establish an efficient method of specifically down-regulating c-MYC expression in MB, we used a siRNA approach. Transfection of human MB cells with siRNA directed against c-MYC resulted in a pronounced down-regulation of c-MYC mRNA expression to 21% in DAOY wt (p < 0.001), 22% in DAOY V11 (p < 0.001), 6% in DAOY M2 (p < 0.001), 33% in D341 (p < 0.001), and 65% in D425 (p = 0.017) compared with negative control siRNA-transfected cells, as determined by quantitative RT-PCR at 48 h following transfection (Figure 1A). At the protein level, transfection of human MB cells with c-MYC siRNA resulted in a significant down-regulation of c-MYC protein expression compared with negative control siRNA-transfected cells as determined by Western blotting 72 h post transfection in all MB cell lines tested (Figure 1B). Transfection of human MB cells with c-MYC siRNA resulted in a significant reduction of c-MYC binding activation to 10% in DAOY wt (p = 0.015), 28% in DAOY V11 (p = 0.009), 21% in DAOY M2 (p = 0.008), 74% in D341 (p = 0.05), and 63% in D425 (p = 0.03), compared with MB cells transfected with negative control siRNA at 72 h following transfection (Figure 1C).

### siRNA-mediated c-MYC down-regulation reduces cellular proliferation, inhibits clonogenic growth, induces G1 cell cycle arrest and CDK inhibitor p21^(waf1/Cip1) ^expression in MB cells

**Figure 2:**
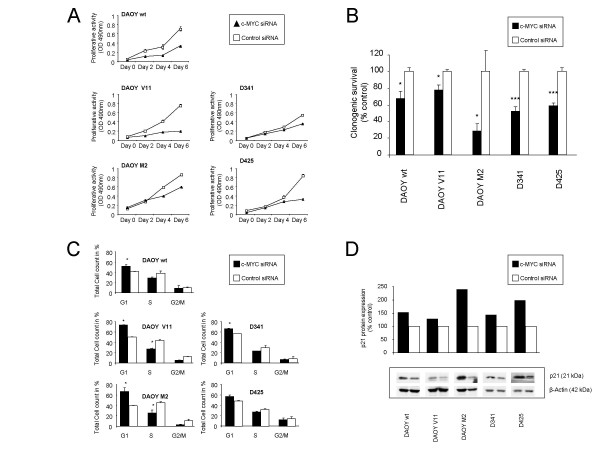
**Knockdown of c-MYC reduces MB cell growth, induces G1 cell cycle arrest and CDK inhibitor p21 ^(waf1/Cip1)^expression**. c-MYC siRNA-transfected cells show reduced cell viability over time and inhibition of clonogenic survival compared with the control siRNA-transfected cells as determined by the MTS assay **(A) **and by a clonogenic survival assay **(B)**. Cell cycle analysis of control and c-MYC siRNA-transfected cells harvested after 48 h by trypsinization, fixed in ethanol and stained with propidium iodide as described in Methods. A total number of 30'000 events per sample were counted. Results are presented as the mean percentages of cells in G1, S, and G2/M of two independent experiments (± standard deviation) **(C)**. c-MYC siRNA-mediated up-regulation of CDK inhibitor p21 ^(waf1/Cip1) ^expression compared with control siRNA-transfected cells as determined by Western blotting and photometrical quantification at 72 h post transfection. Values represent the percentage of p21 ^(waf1/Cip1)^relative to control siRNA-transfected cells **(D)**. * Indicates a significant difference compared to control values, as determined by the Student's t-test (***: P < 0.001, **: P < 0.01,*: P < 0.05).

To test whether siRNA-mediated reduction of c-MYC alters cellular proliferation of human MB cells, we assessed cell viability at 0, 2, 4, and 6 days following c-MYC siRNA or control siRNA transfection. When compared with control siRNA-transfected cells, growth was lower in c-MYC siRNA-transfected MB cell lines (Figure 2A). This effect was independent of basal c-MYC expression. We then determined clonogenic survival using MB cells transfected with control or c-MYC siRNA. After 48 h of exposure to either c-MYC siRNA or to control siRNA, the siRNA was removed, and clonogenic survival was determined. When compared with control siRNA-transfected cells, the clonogenic survival of c-MYC siRNA-transfected cells was significantly reduced, as observed by decreased number of large colonies (colonies with more than 50 cells), to 67% in DAOY wt (p = 0.011), 78% in DAOY V11 (p = 0.026), 26% in DAOY M2 (p = 0.038), 52% in D341 (p = 0.003), and 59% in D425 (p = 0.001) human MB cells (Figure 2B). We then examined whether alterations in the cell cycle underlie the c-MYC siRNA-mediated effect on proliferation of MB cells. Flow cytometric analysis of cells transfected with c-MYC siRNA for 48 h showed consistent changes in the cell cycle distribution compared with control siRNA-transfected cells. In all cell lines tested, there was an increase in the number of cells in the G0/G1 phase, which was significant in DAOY wt (p = 0.028), DAOY V11 (p = 0.001), DAOY M2 (p = 0.033), and D341 (p = 0.006), but slightly below the significance level in D425 (p = 0.062) (Figure 2C). In all MB cells under study there was a decrease in the number of cells in the S-phase, which was significant in DAOY V11 (p = 0.005) and DAOY M2 (p = 0.05), compared with control siRNA-transfected cells. The observation that c-MYC siRNA transfection resulted in an up-regulation of CDK inhibitor p21 ^(waf1/Cip1) ^expression compared with control siRNA-transfected cells to 153% in DAOY wt, 128% in DAOY V11, 239% in DAOY M2, 142% in D341, and 198% in D425, as determined by Western blotting and photometrical quantification (Figure 2D), was in accordance with the G1 arrest induced by c-MYC down-regulation. Taken together, these results demonstrate that down-regulation of c-MYC inhibits tumor cell growth at least partly by induction of cell cycle arrest

### siRNA-mediated c-MYC down-regulation reduces apoptotic cell death in MB cells

**Figure 3:**
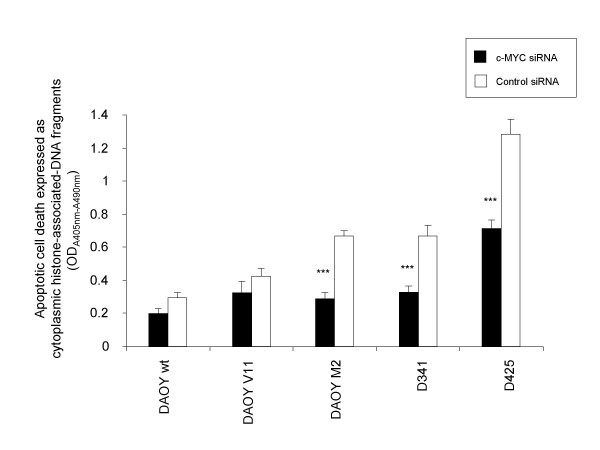
**c-MYC siRNA-mediates a reduction in apoptotic cell death**. Reduced apoptotic levels in cells transfected with c-MYC siRNA compared with control siRNA-transfected cells at 72 h post transfection. Values represent the mean absorbance of cytoplasmatic histone-associated DNA fragments (representative of two independent experiments, (n = 3; ± SD)). * Indicates a significant difference compared to control values, as determined by Student's t-test (***: P < 0.001, **: P < 0.01,*: P < 0.05).

To investigate whether the decrease in cellular growth induced by c-MYC down-regulation was also caused by increased levels of apoptosis, we determined apoptotic cell death in siRNA-transfected MB cells (72 h). This analysis revealed that down-regulation of c-MYC resulted in a reduction of apoptosis in all human MB cell lines tested (Figure 3; DAOY wt: 79% (p = 0.12); DAOY V11: 76% (p = 0.10); DAOY M2: 48% (p = 0.001); D341: 49% (p = 0.002), D425: 54% (p = 0.001)). This inhibitory effect on apoptosis was more pronounced in human MB cell lines expressing high levels of c-MYC. Cell cycle analysis of control and c-MYC siRNA-transfected cells revealed a decrease in the proportion of cells in sub-G1 when exposed to c-MYC siRNA to 74% in DAOY, to 50% in DAOY V11, to 47% in DAOY M2, to 48% in D341, and to 53% in D425 MB cells.

### siRNA-mediated c-MYC down-regulation reduces hTERT mRNA expression and telomerase activity in MB cells

**Figure 4:**
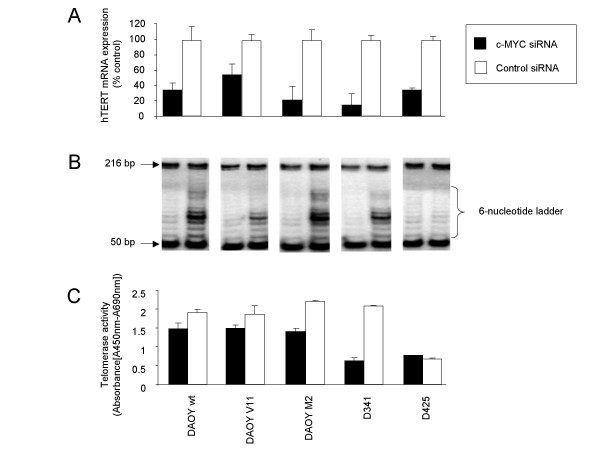
**c-MYC siRNA-mediates inhibition of hTERT mRNA expression and telomerase activity**. Inhibition of hTERT mRNA expression **(A) **and telomerase activity **(B, C) **by c-MYC down-regulation as determined by quantitative RT-PCR **(A)**, by the TRAP assay showing typical products of telomerase activity (6-nucleotide telomerase product ladder) with bands starting from 50 bp and the internal control (IC) in a single band of 216 bp **(B)**, and by the TeloTAGGG telomerase polymerase chain reaction (PCR) enzyme-linked immunosorbent assay (ELISA) kit **(C)**. Values represent the percentage of hTERT mRNA (n = 3; ± SD) and mean absorbance (n = 3; ± SD) of control and c-MYC siRNA-transfected cells **(A, C)**.

To investigate the effects of c-MYC down-regulation on telomerase in MB cells, we determined hTERT mRNA expression by using quantitative RT-PCR at 48 h following transfection with c-MYC siRNA or control siRNA. We found a significant reduction of hTERT mRNA expression in c-MYC siRNA-transfected cells to 34% in DAOY wt, 54% in DAOY V11, 21% in DAOY M2, 15% in D341, and 35% in D425 compared with negative control siRNA-transfected cells, as determined by real-time RT-PCR (Figure 4A). We then determined the effect of c-MYC down-regulation on the enzymatic activity of telomerase and performed a modified TRAP assay on cell lysates 72 h post transfection. This analysis revealed that telomerase activity was inhibited in c-MYC siRNA-transfected DAOY wt, DAOY V11, DAOY M2, and D341 MB cells (Figure 4B). Telomerase activity was found to be reduced to 77% in DAOY wt, 79% in DAOY V11, 63% in DAOY M2, and 30% in D341, compared with negative control siRNA-transfected cells (Figure 4C). In D425 MB cells, which are characterized by low TRAP activity [40], c-MYC inhibition did not alter the telomerase activity (Figure 4B,C).

**Figure 5:**
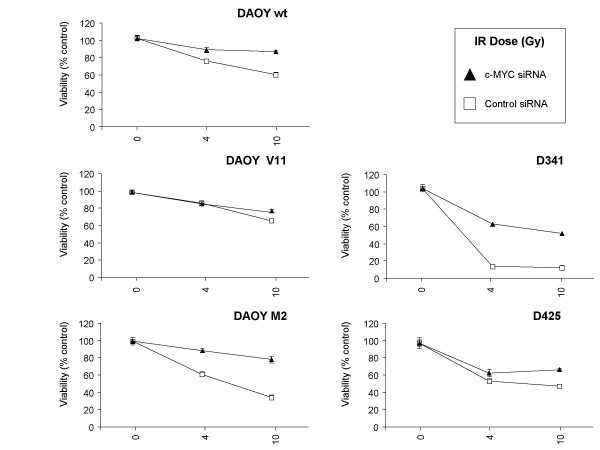
**c-MYC siRNA-mediates reduced susceptibility to ionizing radiation**. Cytotoxicity induced by irradiation in c-MYC siRNA and control siRNA-transfected cells was determined by the MTS assay. Values represent the mean percentage of survival ± SD (n = 3) compared to non-irradiated c-MYC siRNA or control siRNA-transfected cells, respectively. (n = 3; ± SD).

### siRNA-mediated c-MYC down-regulation decreases radio-sensitivity of MB cells

DAOY, D341, and D425 human MB cells transfected with c-MYC or control siRNA were irradiated with 0, 4, and 10 Gy and cell viability was quantified after 72 h. D341 and D425 were more sensitive to IR than DAOY cells harboring mutant p53 [41] (Figure 5). DAOY M2 cells engineered to express higher c-MYC levels were more susceptible to IR than DAOY wt or DAOY V11 (Figure 5). Compared with control siRNA-transfected cells, c-MYC siRNA-transfected cells were more resistant to killing by IR (Figure 5).

### siRNA-mediated c-MYC down-regulation decreases chemo-sensitivity of MB cells

**Figure 6:**
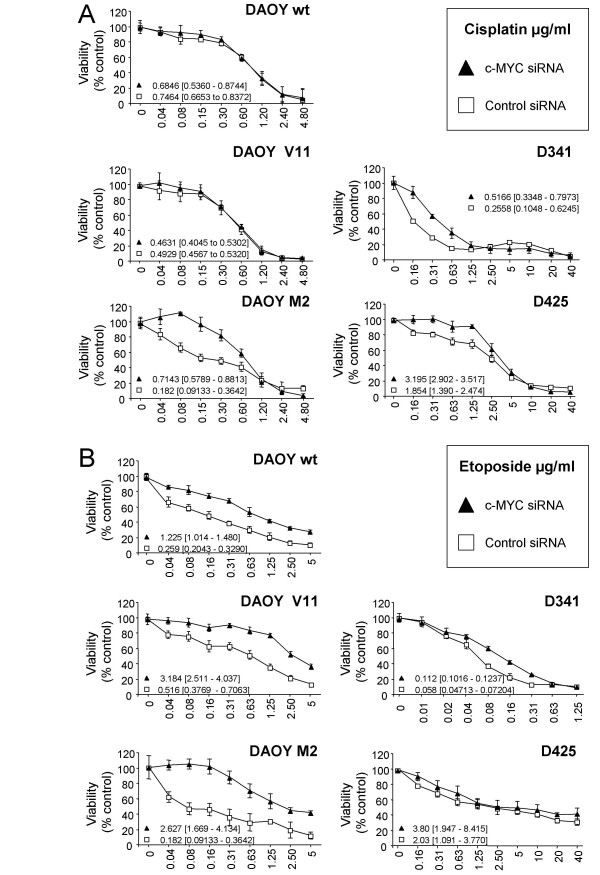
**c-MYC siRNA-mediates reduced susceptibility to cisplatin (A) and etoposide (B) treatment**. Cytotoxicity induced by etoposide and cisplatin in c-MYC siRNA and in control siRNA-transfected cells was determined by the MTS assay. Values represent the mean percentage of survival compared to control cells (n = 3; ± SD). The IC_50 _values and their 95% confidence intervals were calculated from the regression curve and are indicated for each data set.

To investigate whether siRNA-mediated down-regulation of c-MYC alters chemo-sensitivity of MB cells, we measured the cytotoxic response to etoposide and cisplatin. In the case of cisplatin treatment, c-MYC siRNA transfection resulted in a significant decrease in chemo-sensitivity in DAOY M2 (p < 0.001), D425 (p = 0.003), and D341 (p = 0.013) (Figure 6A). However, no changes in chemo-sensitivity were observed in DAOY wt and DAOY V11 human MB cells (Figure 6A). In the case of etoposide treatment, c-MYC siRNA transfection resulted in a significant decrease in chemo-sensitivity in DAOY wt (p < 0.0001), DAOY V11 (p < 0.0001), DAOY M2 (p < 0.0001), and D341 (p < 0.001), and a minor decrease in D425 (p = 0.12) (Figure 6B).

### siRNA-mediated c-MYC down-regulation attenuates the cellular apoptotic response of DAOY MB cells to chemotherapy that is associated with reduced caspase-9 activity

**Figure 7:**
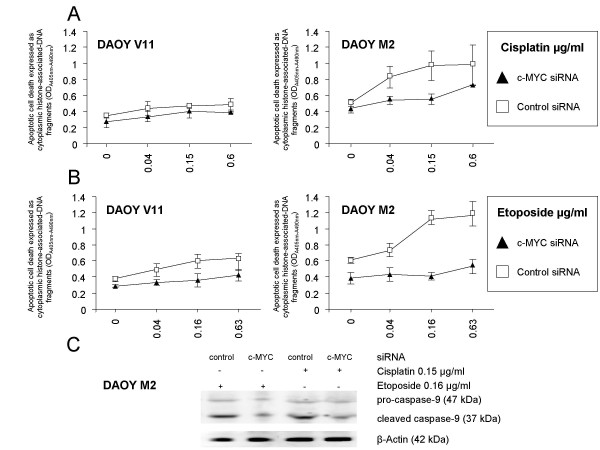
**c-MYC siRNA-mediates inhibition of cisplatin- and etoposide-induced apoptosis (A, B) in DAOY MB cells that is associated with reduced processing of caspase-9(C)**. Minor **(A)** and moderate **(B)** induction of apoptosis in DAOY V11 cells transfected with siRNAs and exposed to cisplatin and etoposide, respectively. Markedly reduced apoptosis in DAOY M2 cells transfected with c-MYC siRNA compared to control siRNA-transfected cells upon cisplatin and etoposide treatment **(A, B)**. Reduced processing of caspase-9 in c-MYC siRNA-transfected DAOY M2 cells after cisplatin and etoposide treatment as assessed by Western blot analysis **(C)**. Actin was used as a loading control. Values represent the mean absorbance of cytoplasmatic histone-associated DNA fragments (representative of two independent experiments, (n = 3; ± SD)).

To investigate whether the reduction of chemo-sensitivity induced by c-MYC down-regulation, as measured by cell viability (Figure 6), was caused by decreased levels of apoptosis, we measured the apoptotic cell death in siRNA-transfected (48 h) DAOY M2 cells – showing significant difference in viability following c-MYC down-regulation and treatment with cisplatin and etoposide – after treatment with either cisplatin or etoposide for 72 h. DAOY V11 was included as a control. Following c-MYC down-regulation, siRNA-transfected DAOY V11 cells were equally sensitive to cisplatin in terms of viability and induction of apoptosis (Figure 6A, 7A). In the case of etoposide treatment, c-MYC siRNA-treated DAOY V11 cells showed reduced cellular sensitivity (Figure 6B) and only a moderate induction of apoptosis compared to control siRNA-transfected cells (Figure 7B). Treatment of control siRNA-transfected DAOY M2 cells with cisplatin and etoposide resulted in a dose-dependent increase of apoptotic cell death when compared with c-MYC siRNA-transfected DAOY M2 cells. Most of the chemotherapeutics, including cisplatin and etoposide [42,43], induce apoptosis mainly through the mitochondrial pathway. Mitochondria-mediated apoptosis involves the cleavage of pro-caspase-9 into the active caspase-9. Next, we examined processing of caspase-9 in DAOY M2 cells, transfected with siRNAs for 48 h and treated for 72 h with cisplatin and etoposide. More caspase-9 cleavage was detected in control siRNA-transfected cells compared to c-MYC siRNA-transfected DAOY M2 cells as determined by Western blotting. This analysis suggests that down-regulation of c-MYC inhibits the apoptotic response to chemotherapy that appears at least partly mediated by caspase-9.

## Discussion

Whereas the impact of c-MYC gene amplification and c-MYC mRNA over-expression on MB histology and prognosis has been investigated intensively [19-24], the biological functions of c-MYC in MB cells remain largely elusive. To our knowledge, the current study represents the first comprehensive analysis of c-MYC targeting in a representative panel of human MB cells expressing different levels of c-MYC.

siRNA-mediated c-MYC down-regulation resulted in an anti-proliferative effect in all human MB cell lines tested. Moreover, the ability of c-MYC siRNA-treated MB cells to form large colonies was significantly reduced. Down-regulation of c-MYC expression resulted in an activation of the CDK inhibitor p21 and in a cell cycle arrest at the G0/G1 phase. These results are supported by findings from others, demonstrating that c-MYC represses the p21 promoter [44], and showing that c-MYC over-expression in primary human fibroblasts results in inhibition of p21 transcription [45]. Moreover, the G1-S progression of eukaryotic cells is in part controlled by c-MYC [2,4,7]. Ectopic expression of c-MYC in quiescent cells promotes the entry into the S-phase [46,47]. On the other hand, down-regulation of c-MYC expression by antisense methods resulted in anti-proliferative effects by preventing S-phase entry [48,49]. Our investigation shows that c-MYC inhibition reduces tumor cell growth suggesting that c-MYC might be an attractive target in MB to inhibit tumor growth.

siRNA-mediated c-MYC down-regulation resulted in a down-regulation of hTERT mRNA expression and in a reduction of telomerase activity in all but D425 human MB cells. D425 cells are characterized by relatively low hTERT mRNA expression and TRAP-negativity [40]. However, the absence of significant correlation between c-MYC and hTERT expression and the difference seen between hTERT mRNA and activity inhibition could be attributed to the presence of several binding sites for other transcription factors – than c-MYC – on hTERT gene promoter and to the fact that expression of hTERT mRNA is not the only regulator of telomerase activity [50-52]. Alternative splicing [53], posttranscriptional modification [54,55], sub-cellular hTERT localization [56] and the presence of enzyme inhibitors may alter telomerase activity. Moreover, it has been suggested that a threshold level of hTERT protein is required in order for telomerase to become active, therefore low levels of hTERT mRNA may not indicate active telomerase [57]. In this respect, hTERT expression has not been found to correlate with telomerase activity in colorectal tumors [58]. Furthermore, hTERT mRNA expression has been detected in lymphocytes regardless of telomerase activity [59] and in some normal telomerase negative tissues such as human brain, prostate liver and ovary [60]. Telomerase impairment can lead to the induction of apoptosis through a mechanism that relies on the inhibition of telomerase catalytic activity and on the consequent telomere shortening [61,62]. However, depending on the telomere length, this mechanism is rather slow. Several generations and a long lag time may be required before telomeres shorten below a critical length [62-64]. Holt et al. have showed that cells with elongated telomeres are more resistant to induction of apoptosis than their parental counterparts with short telomeres [65]. Our experiments were executed in a short time window (72 h) due to cell distress upon treatment with c-MYC siRNA.

DAOY M2 cells engineered to stably over-express c-MYC show higher basal apoptotic activity compared with DAOY wt and DAOY V11 cells, and are characterized by enhanced apoptosis rate *in vivo *[24]. D425 and D341 MB cells (both with c-MYC gene amplification and high c-MYC expression) also show a higher basal apoptotic rate when compared with DAOY wt cells. Moreover, c-MYC inhibition resulted in a significant decrease in the apoptotic rate which was most pronounced in MB cells with high c-MYC expression. It has been shown that c-MYC can drive cells to undergo apoptosis [7-9]. However, this might not be the case for all cell lines. In UW-228 human MB cells, down-regulation of c-MYC by an antisense approach resulted not only in an inhibition of cell growth but also in an increase of apoptosis as determined by FACS analysis [66].

We then analyzed whether c-MYC down-regulation alters sensitivity to IR and chemotherapeutic agents and found a decrease in the sensitivity to IR, cisplatin, and etoposide. This effect was most prominent in cells expressing high c-MYC levels. The differences of cytotoxic effect of cisplatin and etoposide on siRNA-transfected MB cells may be due to the different mode of action that the two drugs exert. The cytotoxicity of cisplatin is mediated by its interaction with DNA to form DNA adducts, which activate several signal transduction pathways and terminate in the activation of apoptosis [67], whereas etoposide belongs to the class of topoisomerase II poison with the ability to stabilize complex between DNA topoisomerase II and DNA that results in a high level of DNA damage [43]. By addressing the question of whether c-MYC alters the response of cells to radio- and chemotherapy, results have been conflicting [68-74]. Our results are in agreement with studies demonstrating that c-MYC sensitize a variety of cells to different cytotoxic treatments [68-70], and that absence of c-MYC expression might confer resistance to anti-cancer agents [71]. However, this effect of c-MYC also seems to be cell type-specific. In human melanoma cells, a decrease in c-MYC expression has been reported to enhance the effect of IR [72] and cisplatin treatment [73,74].

Cancer chemotherapies induce apoptosis [42]. Changes that decrease the ability to activate the apoptotic machinery might affect the sensitivity of cells to a range of chemotherapeutic agents. In order to evaluate whether c-MYC down-regulation affects the susceptibility to apoptosis, we investigated the apoptotic response of siRNAs-transfected DAOY M2 cells following cisplatin and etoposide treatment. This cell line was used, because c-MYC inhibition caused significant reduction of chemo-sensitivity to cisplatin and etoposide. This analysis revealed that down-regulation of c-MYC attenuated drugs-induced apoptosis that was associated with a reduced caspase-9 processing. These results are consistent with findings from other groups showing that c-MYC inhibition mediates resistance to chemotherapy-induced apoptosis by preventing activation of caspase-mediated pathways [68,69,71].

## Conclusion

Taken together, our data show for the first time that targeting c-MYC in human MB cells decreases cell growth, induces cell cycle arrest and reduces telomerase activity. However, c-MYC down-regulation also causes decreased apoptosis and resistance towards IR, cisplatin, and etoposide. Following *in vivo *validation, targeting c-MYC might represent a promising strategy to reduce tumor growth in pretreated MB patients whose tumors have ceased to respond to chemotherapy. Our data also indicate that targeting c-MYC might not be used concomitantly with chemotherapy or radiotherapy in MB because of the risk of reducing the effectiveness of these therapies.

Emerging evidence indicates that the different precursor cell populations that form the cerebellum and the cell signaling pathways that regulate its development likely represent distinct compartments from which the variable subtypes of MB arise [75,76]. It remains to be tested whether down-regulation of c-MYC has MB subtype specific effects or is rather MB subtype independent.

## Competing interests

The authors declare that they have no competing interests.

## Authors' contributions

AOVB performed most experimental work including data analysis and he drafted the manuscript. TS participated in the study design and supervised the experimental work. AOVB and COJ carried out together the irradiation experiments under the supervision of MP. DS and CGE stably transfected the DAOY cell lines and participated in study design and data analysis.  LA contributed in some experiments. MAG guided the study and finalized the manuscript. AA helped by finalizing the manuscript. All authors read and approved the final manuscript.

## Pre-publication history

The pre-publication history for this paper can be accessed here:

http://www.biomedcentral.com/1471-2407/9/10/prepub
